# Dislocation of the First Metacarpophalangeal Joint Complicated by the Interposition of the Thumb Sesamoid Bones: A Case Report

**DOI:** 10.7759/cureus.72950

**Published:** 2024-11-03

**Authors:** Matthias Barden, Maritza Lozano, Jacob R Bosley

**Affiliations:** 1 Undergraduate Medical Education, School of Medicine, University of California Riverside, Riverside, USA; 2 Emergency Medicine, Eisenhower Health, Rancho Mirage, USA; 3 Orthopedic Surgery, Eisenhower Health, Rancho Mirage, USA

**Keywords:** hand x-rays, locked thumb syndrome, metacarpophalangeal joint dislocation, orthopedic injury, sesamoid bones

## Abstract

We present a case of a thumb metacarpophalangeal (MCP) joint dislocation complicated by the interposition of the sesamoid bones. This case highlights a clinical scenario referred to as the “locked thumb” syndrome, in which a first-digit MCP dislocation is complicated by an entrapped anatomical structure that hinders closed reduction. In this case, the thumb sesamoid bones became lodged at the base of the dislocated proximal phalanx. Due to this mechanical blockage, bedside closed reduction failed, and operative management was required.

## Introduction

Dislocation of the thumb metacarpophalangeal (MCP) joint is a relatively rare injury that generally occurs with forced hyperextension, such as during a fall onto an outstretched hand [1]. Uncomplicated dorsal thumb MCP dislocations can usually be reduced at the bedside [2]. However, a significant portion of these cases can be complicated by a mechanical blockage that prevents closed reduction [3,4]. Multiple different anatomical and pathological structures have been implicated in causing this clinical scenario of the "locked thumb." We present a case of a thumb MCP dislocation that was complicated by the interposition of the sesamoid bones, causing closed reduction to fail and requiring operative management. 

## Case presentation

**Figure 1:**
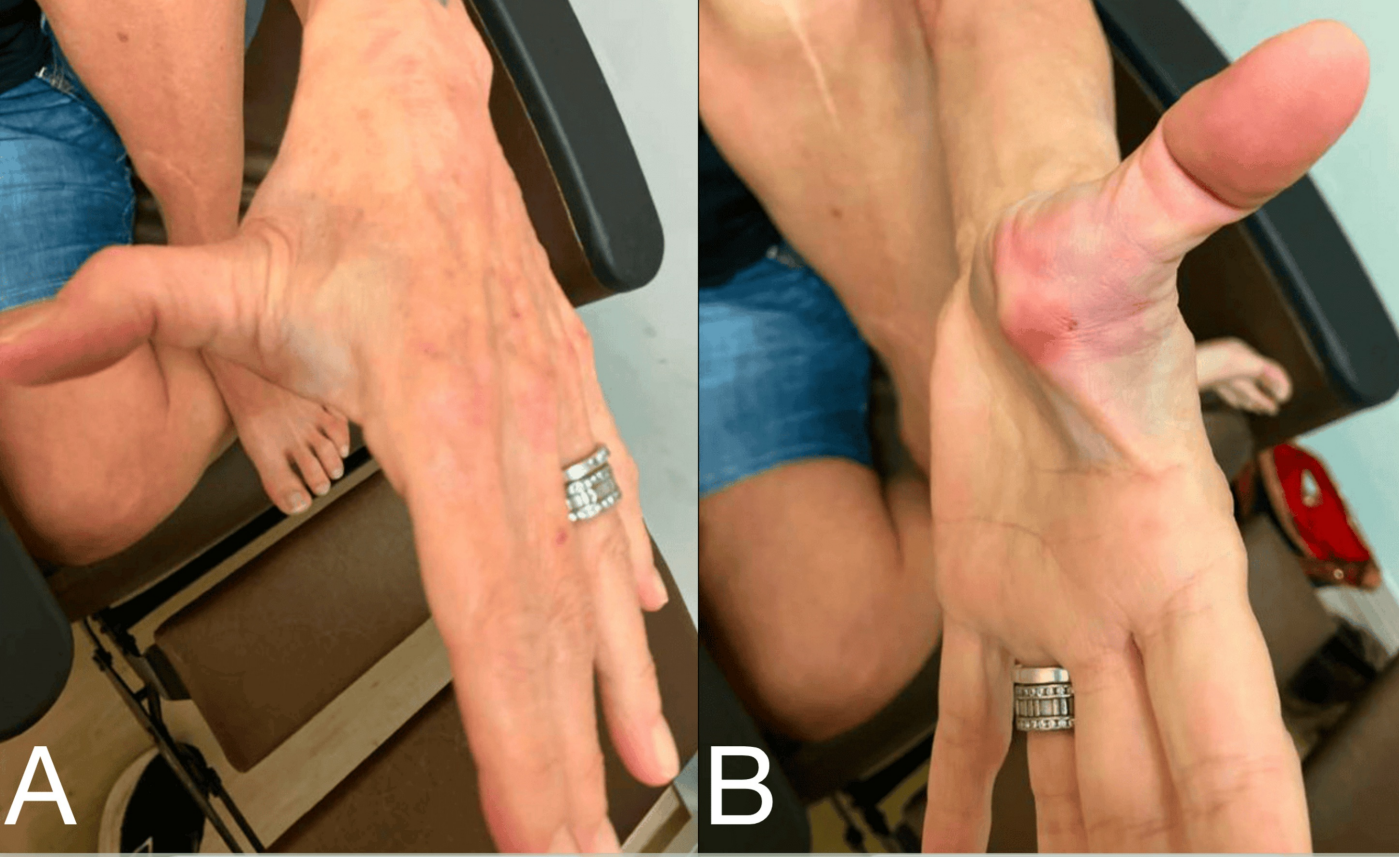
Clinical photographs of the left thumb deformity with apex volar angulation of the first MCP joint. (A) Dorsal view. (B) Volar view. MCP: metacarpophalangeal.

A 67-year-old right-hand-dominant female patient presented to our emergency department with an injury to the left thumb after a fall. She was out walking her dog when she became tangled in the leash causing her to fall forward onto her outstretched hand. After the fall she noted deformity and loss of mobility of the left first MCP joint, causing her to seek care in the emergency department (Figure 1). On exam, she was found to be distally neurovascularly intact and was without injury or complaint otherwise. 

**Figure 2:**
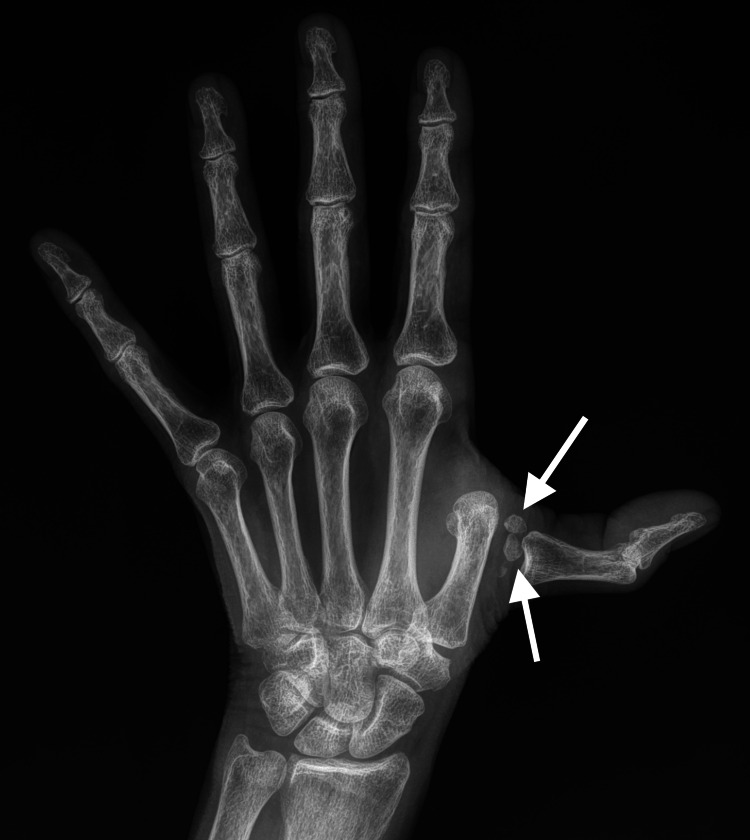
Radiograph of the patient's left hand demonstrating a dorsally displaced dislocation of the first metacarpophalangeal joint with interposition of multiple sesamoid bones. The white arrows indicate the sesamoid bones lodged at the base of the dislocated first proximal phalanx.

Radiographs of the hand were obtained, which identified a dorsal dislocation of the first MCP joint with interposition of the sesamoid bones (Figure 2).

**Figure 3:**
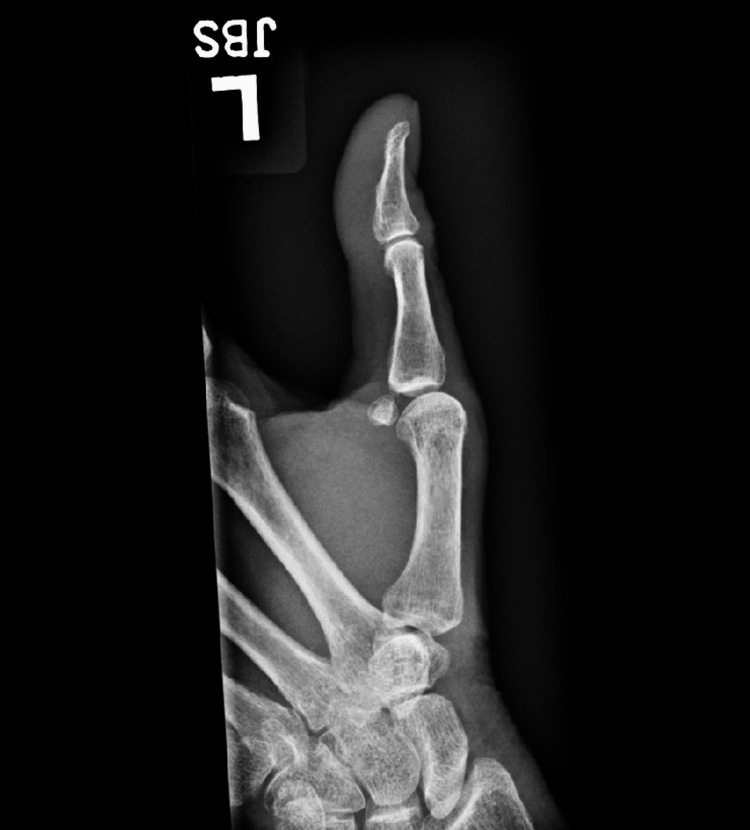
Post-reduction radiograph showing restoration of the normal positioning of the MCP joint and sesamoid bones. MCP: metacarpophalangeal.

Closed reduction was attempted in the emergency department without success. Given the complex nature of her dislocation, she was subsequently taken to the operating room for open reduction. In the operating room, MCP dislocation with interposition of the sesamoids was confirmed without any associated ligamentous injury. After the surgery, the patient was immobilized by a thumb spica splint. She was doing well on revaluation at her two-week follow-up appointment, with intact tendon exam function on examination. There was some ongoing mild pain, worsened by range of motion movements. She reported some numbness on the volar-radial aspect of the thumb as well. Repeat radiographs were obtained at that time (Figure 3). She was transitioned to a removable splint as needed for comfort and was provided instruction on sensory and range of motion rehabilitation exercises.

## Discussion

MCP dislocations are considered rare; however, reliable data on the incidence are lacking. The thumb is the most frequently affected digit in MCP joint dislocations, likely due to its greater range of mobility. Dorsal dislocations are much more common than volar dislocations. The locked thumb syndrome refers to a first-digit dorsal MCP dislocation that cannot be reduced without surgery due to mechanical blockage by some entrapped objects. Multiple obstructing structures have been implicated in cases of locked thumb, including fracture fragments, osteophytes, gouty tophi, and volar plate ligaments [5]. A frequent cause of locked thumb syndrome is entrapment of the thumb sesamoid bones within the dislocated joint. Despite variable expressions of the sesamoids at other anatomical locations, the thumb sesamoid bones seem to be nearly uniformly present [6,7]. These sesamoids are anatomically embedded within the volar plate ligaments, so these ligaments are likely involved in the locking mechanism of the intrapositioned sesamoid bones, even in the absence of a ligament rupture [8,9]. There are multiple similar cases reported in the medical literature, but we find that the radiograph presented here is an excellent example that clearly demonstrates the phenomenon [10,11]. Clinicians should recognize the potential for interposition of the sesamoid bones or other causes of mechanical obstruction that may complicate first MCP joint dislocations. In such cases, closed reduction has a high probability of failure and operative management may well be required [12].

## Conclusions

A locked thumb is a well-described clinical scenario in which a first MCP joint dislocation cannot be reduced at the bedside due to mechanical blockage from an anatomical or pathological structure. This report is provided to call attention to this diagnosis and share an interesting radiograph demonstrating a locked thumb caused by the interposition of the sesamoid bones. Emergency physicians should be aware of the locked thumb phenomenon to help guide the management of complicated first MCP dislocations. Bedside closed reduction is a noninvasive modality that may be attempted, but it is likely to be unsuccessful, so proceeding to emergency department sedation, regional anesthesia, or other invasive interventions should likely be avoided. Radiographs demonstrating the interposition of the sesamoid bones or other structures that have been implicated in the locked thumb syndrome can be a key clinical indicator that specialist consultation for open reduction may well be required. In this case, bedside closed reduction of a dislocated first MCP joint was unsuccessful due to the interposition of the thumb sesamoid bones, and as such, an orthopedic surgeon was consulted and performed successful open surgical reduction.
